# Supplementary figures and images for: Symbiotic bacteria confer insecticide resistance by metabolizing buprofezin in the brown planthopper, Nilaparvata lugens (Stål)
Source: PLoS Pathog. 2023 Dec 13;19(12):e1011828. doi: 10.1371/journal.ppat.1011828 (PMC10718449; doi:10.1371/journal.ppat.1011828)

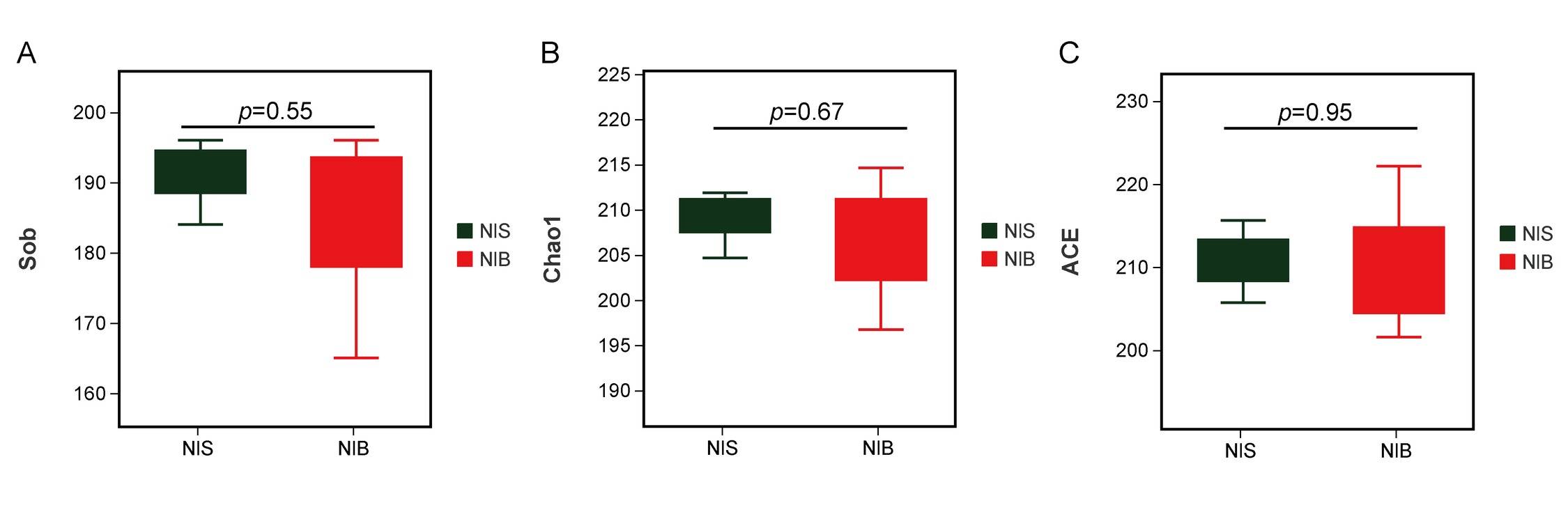

Supplement: S1 Fig — (TIF) [file ppat.1011828.s001.tif]

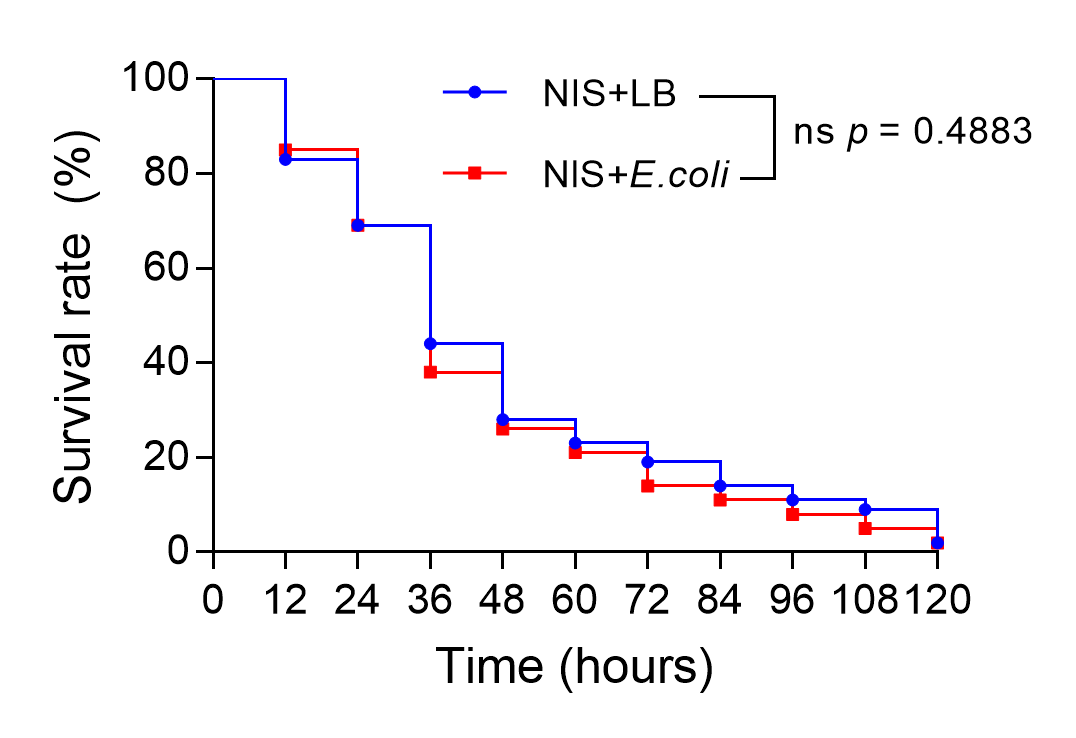

Supplement: S2 Fig — (TIF) [file ppat.1011828.s002.tif]

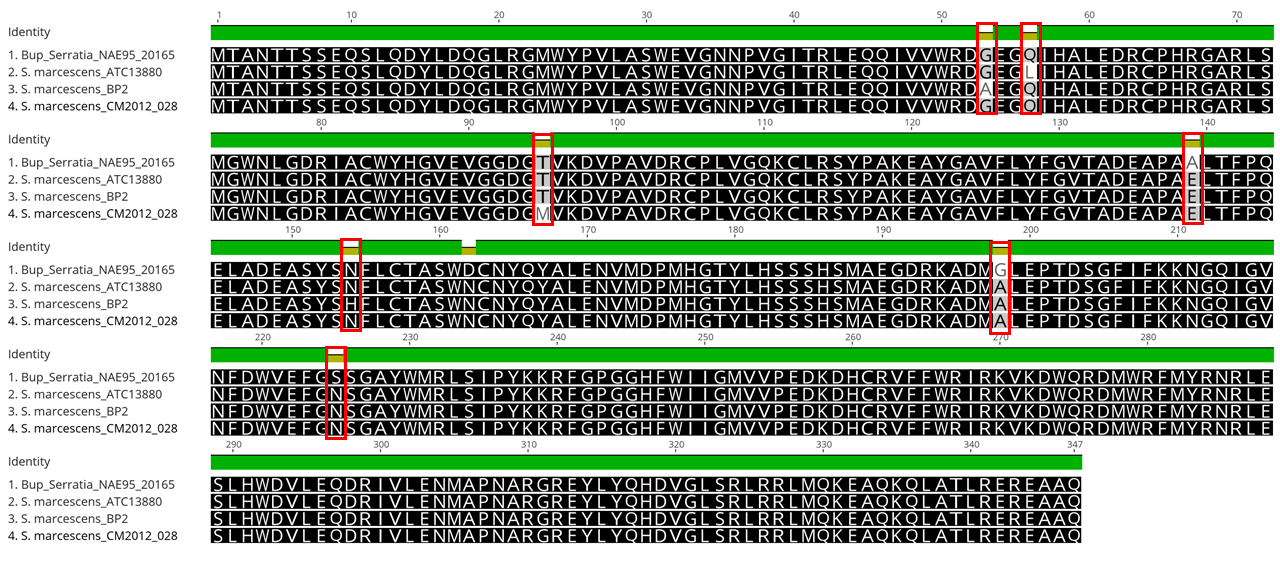

Supplement: S3 Fig — The red boxes circle different amino acides. A total of seven amino acides of NAE95_20165 from Bup_Serratia are inconsistent with other three S. marcescens. (TIF) [file ppat.1011828.s003.tif]

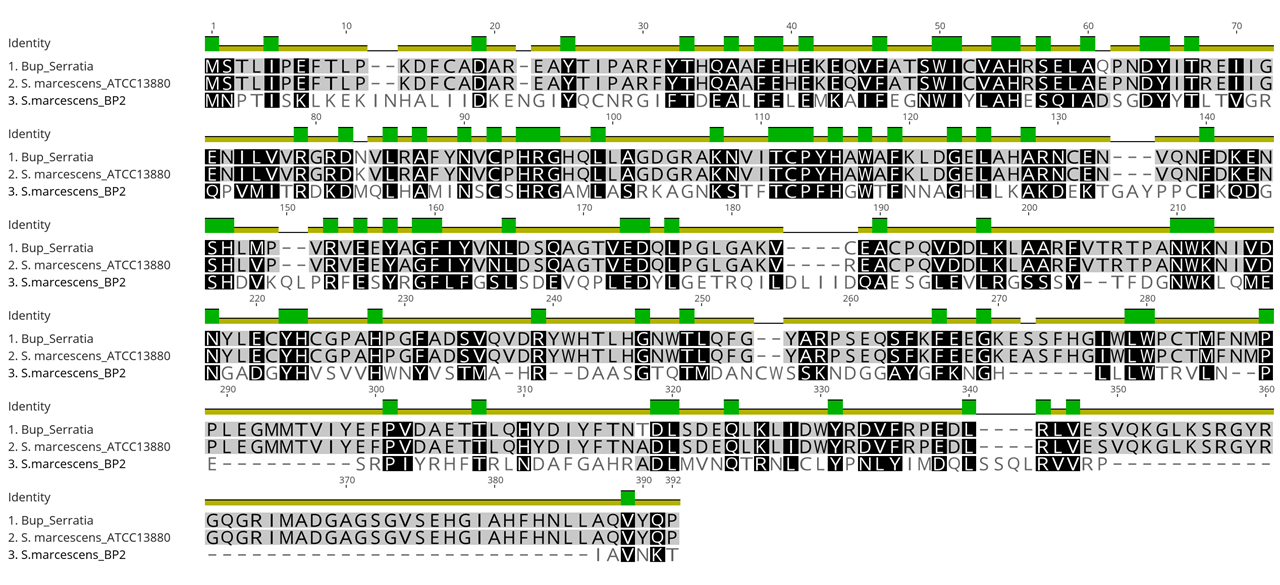

Supplement: S4 Fig — There are very low identities in NAE95_20050 among different S. marcescens strains, even NAE95_20050 is absent on genome of S. marcescens CM2012-028. (TIF) [file ppat.1011828.s004.tif]

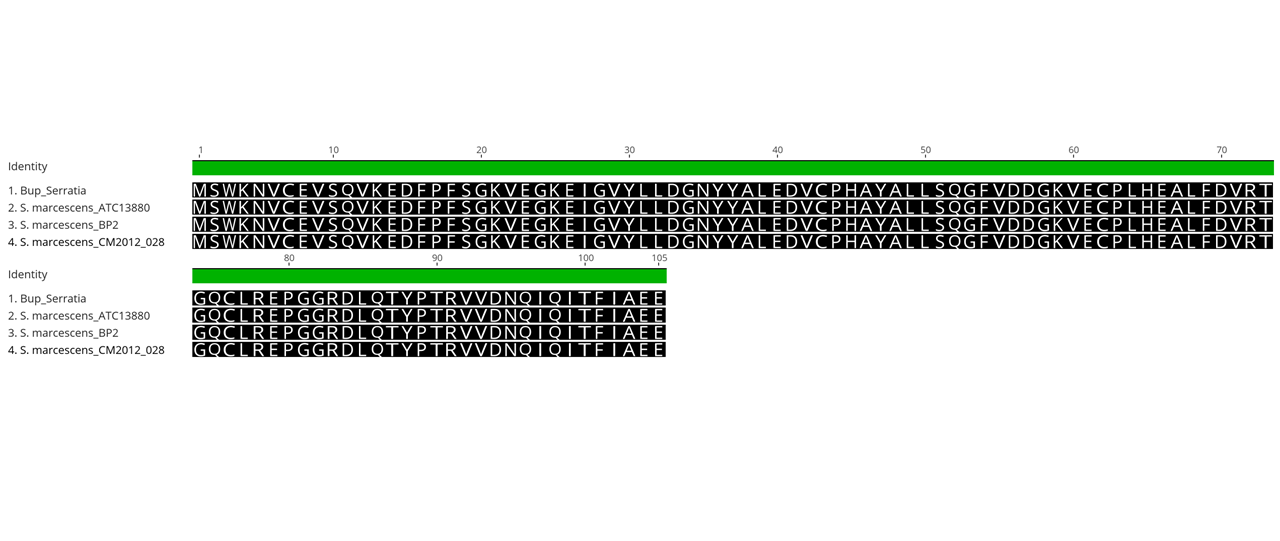

Supplement: S5 Fig — It is completely conserved among different S. marcescens strains. (TIF) [file ppat.1011828.s005.tif]

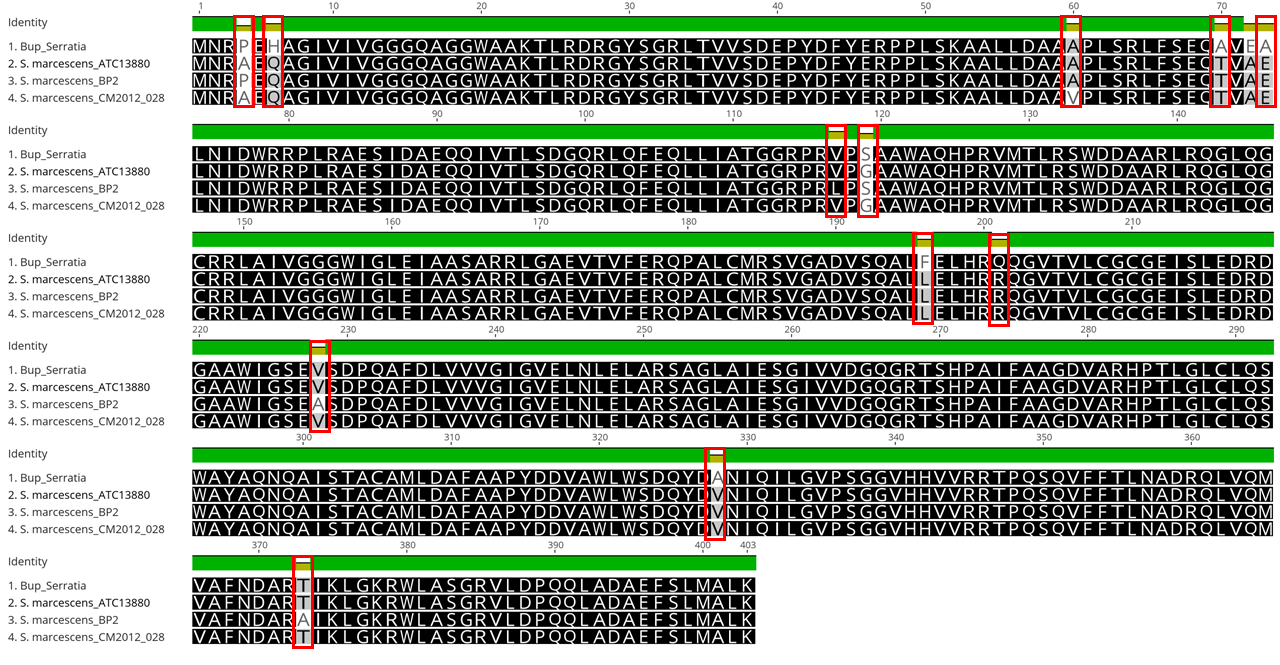

Supplement: S6 Fig — The red boxes circle different amino acides. A total of twelve amino acides of NAE95_20145 from Bup_Serratia are inconsistent with other three S. marcescens. (TIF) [file ppat.1011828.s006.tif]

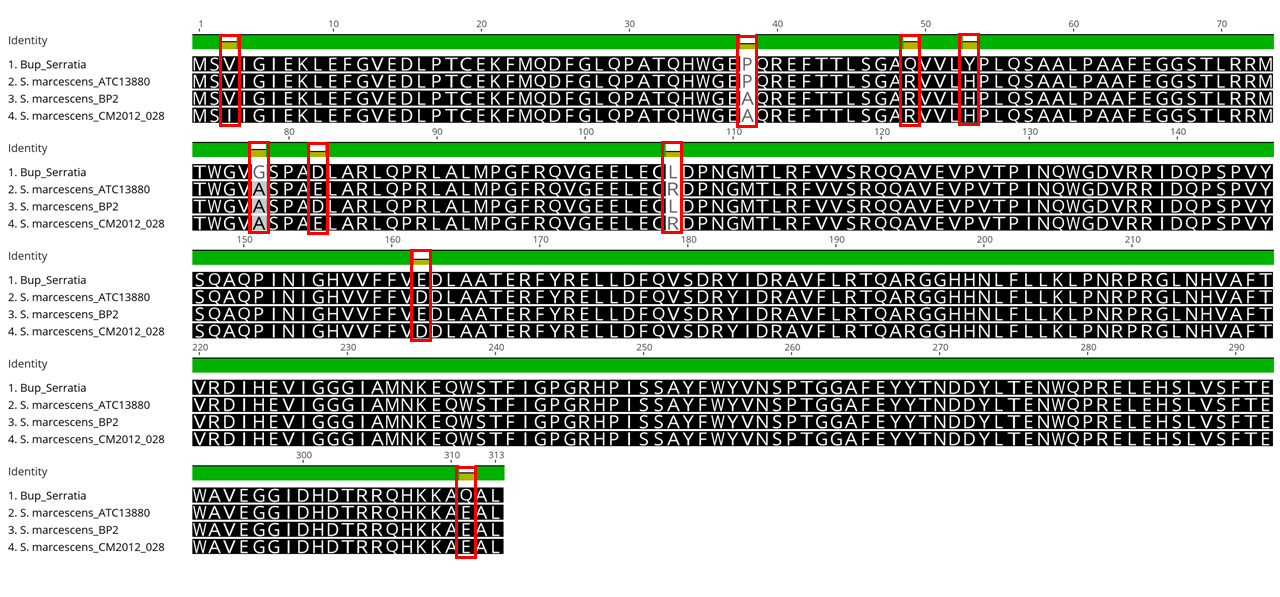

Supplement: S7 Fig — The red boxes circle different amino acides. A total of nine amino acides of NAE95_20150 from Bup_Serratia are inconsistent with other three S. marcescens. (TIF) [file ppat.1011828.s007.tif]

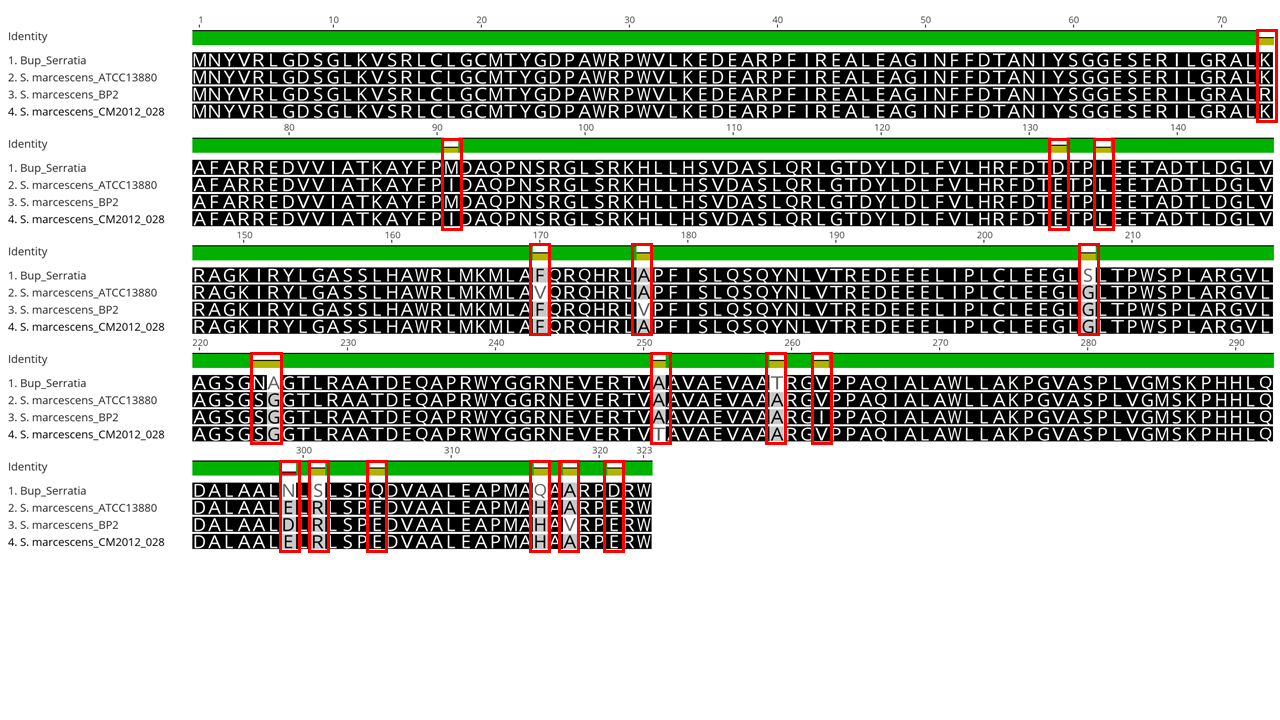

Supplement: S8 Fig — The red boxes circle different amino acides. A total of eighteen amino acides of NAE95_03695 from Bup_Serratia are inconsistent with other three S. marcescens. (TIF) [file ppat.1011828.s008.tif]

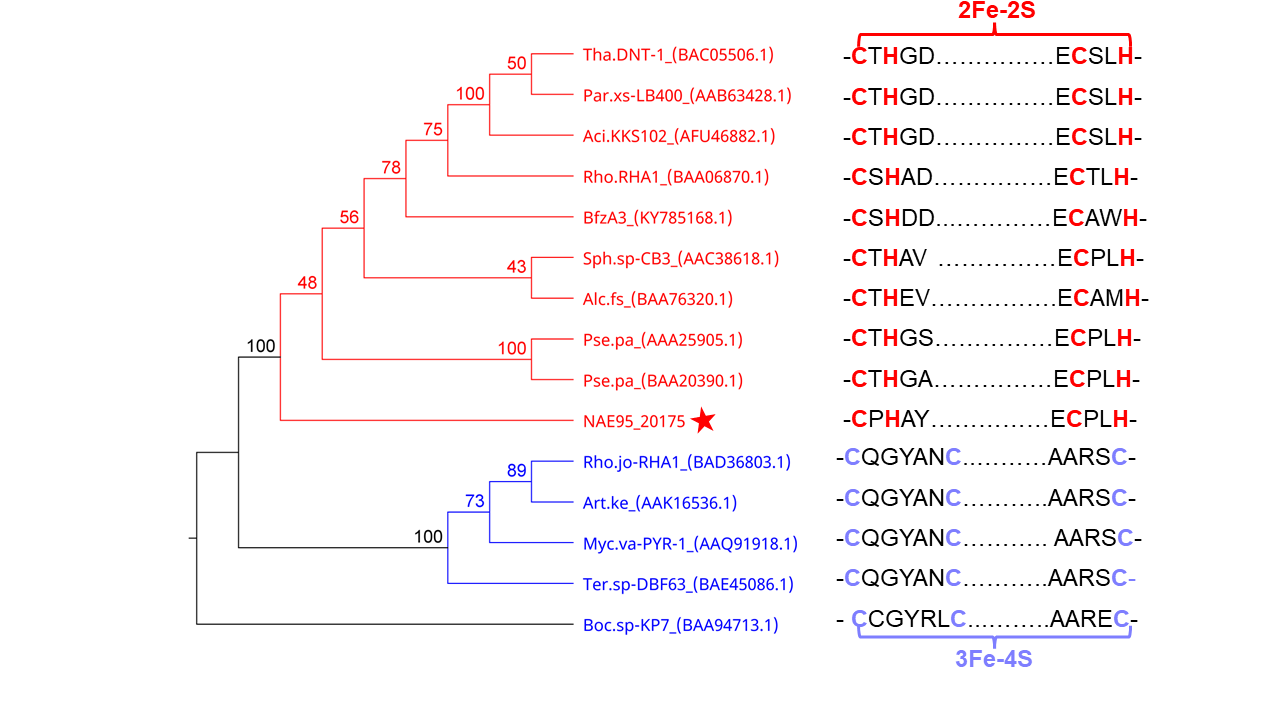

Supplement: S9 Fig — (TIF) [file ppat.1011828.s009.tif]

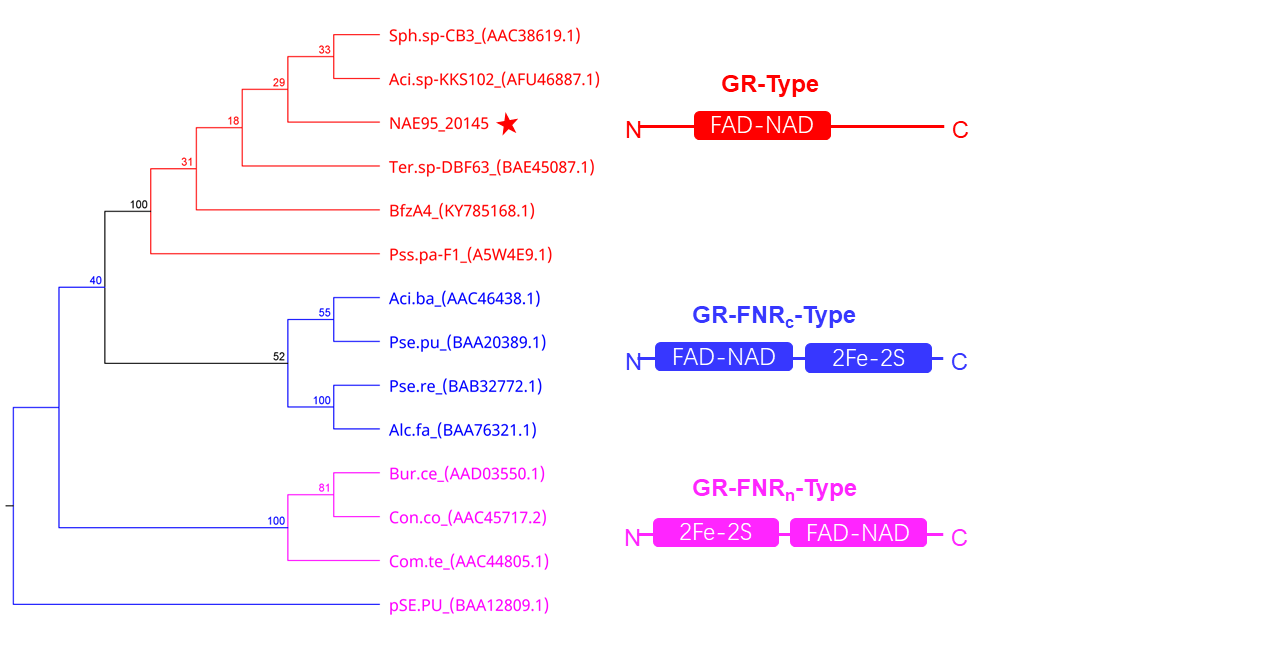

Supplement: S10 Fig — (TIF) [file ppat.1011828.s010.tif]

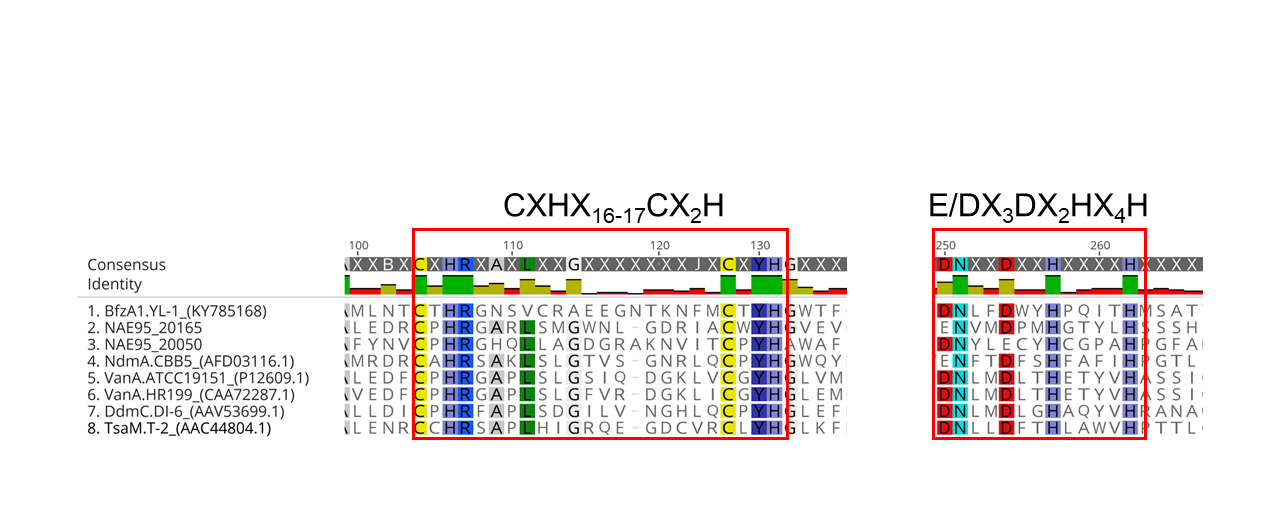

Supplement: S11 Fig — The two red boxes indicate the conserved Rieske [2Fe-2S] center and mononuclear iron-binding site respectively. (TIF) [file ppat.1011828.s011.tif]
