# Supplementary material for: Symbiotic bacteria confer insecticide resistance by metabolizing buprofezin in the brown planthopper, Nilaparvata lugens (Stål)
Source: PLoS Pathog. 2023 Dec 13;19(12):e1011828. doi: 10.1371/journal.ppat.1011828 (PMC10718449; doi:10.1371/journal.ppat.1011828)
Supplement: S1 Table — (DOCX) [file ppat.1011828.s012.docx]

S1 Table. Primers used in this study

| Gene name | Primer sequences | Product size | Amplification efficiency |
| --- | --- | --- | --- |
| rplU | F: GCTTGGAAAAGCTGGACATC | 191 bp | 93.87 % |
|  | R: TACGGTGGTGTTTACGACGA |  |  |
| NAE95_20165 | F: AGTTAACCCCGATCTGTCCG | 153 bp | 95.82 % |
|  | R: ATTTTCTGTGTACCGCCAGC |  |  |
| NAE95_20175 | F: GGGTGGGATAGGTTTGCAGA | 196 bp | 105.98 % |
|  | R: CCGGCAAAGTGGAAGGAAAA |  |  |
| NAE95_20145 | F: AAAGAAAACTCCGCATCCGC | 191 bp | 99.28 % |
|  | R: ATATCCAGATCCTCGGCGTG |  |  |
| NAE95_20150 | F: ACTGCTCTTTGTTCATGGCG | 200 bp | 100 % |
|  | R: ATCGCGAGCTGTTGGATTTC |  |  |
| NAE95_20050 | F: AACGTCATCACCTGCCCTTA | 164 bp | 96.87 % |
|  | R: TGACGTAAATAAACCCGGCG |  |  |
| NAE95_03695 | F: GATCCTGCATGCTGACGAAG | 247 bp | 101.63 % |
|  | R: CGCCAAGATCATGCAATCCA |  |  |
