# Supplementary material for: Symbiotic bacteria confer insecticide resistance by metabolizing buprofezin in the brown planthopper, Nilaparvata lugens (Stål)
Source: PLoS Pathog. 2023 Dec 13;19(12):e1011828. doi: 10.1371/journal.ppat.1011828 (PMC10718449; doi:10.1371/journal.ppat.1011828)
Supplement: S2 Table — (DOCX) [file ppat.1011828.s013.docx]

S2 Table. Collecting information of *N. lugens* field populations

| NO. | Populations | Location (City, Province) | Longitude and latitude | Year |
| --- | --- | --- | --- | --- |
| 1 | SG | Shaoguan, Guangdong | 113.36°E, 24.40°N | 2022 |
| 2 | MM | Maoming, Guangdong | 111.01°E, 21.51°N | 2022 |
| 3 | TH | Taihe, Jiangxi | 114.90°E, 26.78°N | 2022 |
| 4 | SG_1 | Shanggao, Jiangxi | 114.92°E, 28.23°N | 2022 |
| 5 | NN | Nanning, Guangxi | 112.55°E, 28.27°N | 2022 |
| 6 | LJ | Lujing, Anhui | 117.28°E, 31.25°N | 2022 |
| 7 | FQ | Fuqing, Fujian | 119.38°E, 25.72°N | 2022 |
| 8 | NX | Ningxiang, Hunan | 112.55°E, 28.27°N | 2022 |
| 9 | SD | Shaodong, Hunan | 111.74°E, 27.25°N | 2022 |
| 10 | GG | Guigang, Guangxi | 109.34°E, 23.06°N | 2022 |
