# Supplementary material for: Symbiotic bacteria confer insecticide resistance by metabolizing buprofezin in the brown planthopper, Nilaparvata lugens (Stål)
Source: PLoS Pathog. 2023 Dec 13;19(12):e1011828. doi: 10.1371/journal.ppat.1011828 (PMC10718449; doi:10.1371/journal.ppat.1011828)
Supplement: S3 Table — (DOCX) [file ppat.1011828.s014.docx]

S3 Table. Summary of the sequencing data for the microbiota from the NIS and NIB strains of *N. lugens*

| Sample | Raw Reads | Clean Tags | Effective Tags | Effective Ratio (%) | Good’s Coverage |
| --- | --- | --- | --- | --- | --- |
| NIB_1 | 134324 | 132827 | 119601 | 89.04 | 0.9967 |
| NIB_2 | 129988 | 128490 | 115105 | 88.55 | 0.9967 |
| NIB_3 | 133539 | 132047 | 118046 | 88.40 | 0.9970 |
| NIS_1 | 131436 | 130038 | 123110 | 93.67 | 0.9967 |
| NIS_2 | 135144 | 133749 | 128643 | 95.19 | 0.9964 |
| NIS_3 | 131753 | 130207 | 123620 | 93.83 | 0.9968 |
