# Supplementary material for: Symbiotic bacteria confer insecticide resistance by metabolizing buprofezin in the brown planthopper, Nilaparvata lugens (Stål)
Source: PLoS Pathog. 2023 Dec 13;19(12):e1011828. doi: 10.1371/journal.ppat.1011828 (PMC10718449; doi:10.1371/journal.ppat.1011828)
Supplement: S4 Table — (DOCX) [file ppat.1011828.s015.docx]

S4 Table. General features of the genome of *Bup_Serratia*

| Attribute | Value |
| --- | --- |
| Genome size (bp) | 5110189 |
| G+C ratio (%) | 59.47 |
| Protein-coding genes | 4628 |
| rRNA | 22 |
| tRNA | 88 |
| sRNA | 37 |
