# Supplementary material for: Symbiotic bacteria confer insecticide resistance by metabolizing buprofezin in the brown planthopper, Nilaparvata lugens (Stål)
Source: PLoS Pathog. 2023 Dec 13;19(12):e1011828. doi: 10.1371/journal.ppat.1011828 (PMC10718449; doi:10.1371/journal.ppat.1011828)
Supplement: S5 Table — (DOCX) [file ppat.1011828.s016.docx]

S5 Table. Gene information used for phylogenetic tree construction

| Organism designation | Organism | Accession number |
| --- | --- | --- |
| NAE95_20165 | *Bup_Serratia* | OR282763 |
| NAE95_20050 | *Bup_Serratia* | OR282764 |
| NAE95_20175 | *Bup_Serratia* | OR282762 |
| NAE95_20150 | *Bup_Serratia* | OR282766 |
| NAE95_20145 | *Bup_Serratia* | OR282765 |
| NAE95_03695 | *Bup_Serratia* | OR282767 |
| Ser.ma | *Serratia microhaemolytica* | WP_127957074.1 |
| Com.ti | *Comamonas testosteroni* | AAC44804.1 |
| Myc.PYP-1 | *Mycolicibacterium_vanbaalenii* PYP-1 | AAQ91914.2 |
| BfzA1 | *Rhodococcus qingshengii* YL-1 | ASM60826.1 |
| Ra.U2 | *Ralstonia sp.* U2 | AAD12610.1 |
| Sph.sp | *Phingomonas sp.* | CAA51365.1 |
| Es.coil.HS | *Escherichia coli.* HS | A8A344.1 |
| Esc.coil.K-12 | *Escherichia coli* K-12 | P0ABR5.1 |
| Com.ti | *Comamonas testosteroni* | Q46372.1 |
| Pse.pa | *Pseudomonas putida* | 07944.1 |
| Bil.de | *Rhodococcus jostii* RHA1 | BAA06868.1 |
| Pse.pa | *Pseudomonas putida* | BAA20390.1 |
| Pse.pa | *Pseudomonas putida* | AAA25905.1 |
| Sph.sp-CB3 | *Sphingomonas sp*. CB3 | AAC38618.1 |
| Alc.fs | *Alcaligenes faecalis* | BAA76320.1 |
| BfzA3 | *Rhodococcus qingshengii* YL-1 | ASM60824.1 |
| Rho.RHA1 | *Rhodococcus jostii* RHA1 | BAA06870.1 |
| Tha.DNT-1 | *Thauera sp*. DNT-1 | BAC05506.1 |
| Aci.KKS102 | *Acidovorax sp*. KKS102 | AFU46882.1 |
| Par.xs-LB400 | *Paraburkholderia xenovorans* LB400 | AAB63428.1 |
| Pse.rs | *Pseudomonas resinovorans* | BAB32747.1 |
| Pse.fi | *Pseudomonas furukawaii* | P08695.2 |
| Rho.RHA1 | *Rhodococcus jostii* RHA1 | Q9KWQ5.1 |
| Nox.fa | *Nocardia farcinica* | WP_195027493.1 |
| BfzC | *Rhodococcus qingshengii* YL-1 | ASM60828.1 |
| Act.fs | *Actinomadura formosensis* | WP_174548804.1 |
| Dac.se | *Dactylosporangium siamense* | WP_203848825.1 |
| Rho.as | *Rhodococcus aetherivorans* | QRI79080.1 |
| Rho.rr | *Rhodococcus ruber* | UQB75379.1 |
| Rho.rr | *Rhodococcus ruber* | MBD8056806.1 |
| Pha.cm | *Phanerodontia chrysosporium* | Q01752.1 |
| Chl.bm | *Chloroflexota bacterium* | RLT53382.1 |
| Rey.sp | *Reyranella sp.* | MBR2814464.1 |
| Pse_sp.K10HN5 | *Salmonella enteritidis* | MMH97360.1 |
| Amy.pi | *Amycolatopsis pithecellobii* | WP_154757868.1 |
| Rho.ps | *Rhodococcus pyridinivorans* | MCD5422702.1 |
| Noc.fa | *Nocardia farcinica* | MBF6258380.1 |
| BfzB | *Rhodococcus qingshengii* YL-1 | ASM60831.1 |
| Par.xs-LB400 | *Paraburkholderia xenovorans* LB400 | AAB63429.1 |
| Pss.pa-F1 | *Pseudomonas putida* F1 | A5W4E9.1 |
| Rho.ji-RHA1 | *Rhodococcus jostii* RHA1 | BAD36804.1 |
| Arr.ki | *Arthrobacter keyseri* | AAK16537.1 |
| Ter.sp-DBF63 | *Terrabacter sp*. DBF63 | BAE45087.1 |
| BfzA4 | *Rhodococcus qingshengii* YL-1 | ASM60825.1 |
| Noc.sp-KP7 | *Nocardioides sp*. KP7 | BAA94714.1 |
| Sph.sp-CB3 | *Sphingomonas sp*. CB3 | AAC38619.1 |
| Aci.sp-KKS192 | *Acidovorax sp*. KKS102 | AFU46887.1 |
| Sph.sp | *Sphingomonas sp*. | CAA05635.1 |
