# Supplementary material for: Symbiotic bacteria confer insecticide resistance by metabolizing buprofezin in the brown planthopper, Nilaparvata lugens (Stål)
Source: PLoS Pathog. 2023 Dec 13;19(12):e1011828. doi: 10.1371/journal.ppat.1011828 (PMC10718449; doi:10.1371/journal.ppat.1011828)
Supplement: Attachment — Submitted filename: Response letter-PPATHOGENS-D-23-01409 .docx [file ppat.1011828.s017.docx]

Dear Dr McGraw,

Thank you for your letter dated 6-Oct-2023 regarding our manuscript entitled “Symbiotic bacteria confer insecticide resistance by metabolizing buprofezin in the brown planthopper, *Nilaparvata lugens* (Stål)” (PPATHOGENS-D-23-01409). We appreciate the reviewers’ comments and suggestions on our manuscript.

We have now carefully revised our manuscript according to reviewers’ comments and suggestions. In the following, we list our point-by-point explanations in response to the reviewers’ comments. In our revised manuscript, we use red text to show where we have made the changes.

We would like to take this opportunity to thank you for your consideration of our manuscript for publication in your journal. We believe that we have addressed all the comments raised by the reviewers. If you have any questions about this manuscript, please don’t hesitate to contact me.

Sincerely,

Congfen Gao, Ph.D

Part II – Major Issues: Key Experiments Required for Acceptance

Reviewer #1: I don't see any major issues regarding this study. However, I feel that there is room to improve the clarity of the manuscript as noted below:

**Response**: Thank you for your kind comments and suggestions. We have carefully revised our manuscript according to your advice. And we hope that you find this version acceptable.

1) The resistant strain originally collected from the field was 44.3-fold resistance to buprofezin as compared with the susceptible strain. After the laboratory selections with buprofezin for five generations, what was the level of buprofezin resistance of the insect when it was used in the study?

**Response**: Many thanks for your comment. It is regretful that this experiment was finished in 2021 when our primary aim was to enrich any possible symbiotic bacteria that can degrade buprofezin in bodies of BPH by treating with buprofezin. Thus we did not estimate the LC_50_ values after the treatment of the BPH strain for five generations with buprofezin. However, we could confirm that the resistance level of buprofezin was increased from the change in survival rate.

2) The authors mentioned "where buprofezin was used as the sole carbon source" Lines 200-201). This statement may be true when the authors only considered the chemical added to the LB medium; however, the statement seems to have ignored the carbon source from the LB medium, which the bacteria can use for them to grow. In comparison to the small amount of buprofezin in the medium, the major carbon source for the bacteria is the LB medium.

**Response**: Many thanks for your comment. As mentioned in the methods of lines 191-192, Mineral salts medium (MM) only contained K2HPO4, KH2PO4, NH4NO3, MgSO4·7H2O, NaCl and water. This carbon-free mineral salts medium did not contain LB medium. MIM requires a carbon source to be added before it can sustain bacterial growth. Here, MM containing buprofezin (BMM) was used to enrich the bacteria that can degrade buprofezin, so buprofezin was the sole carbon source.

3) From the evolution point of view, I don't think that this particular symbiotic bacterial strain must rely on buprofezin as the sole carbon source for its growth as buprofezin as an insecticide is very new to the bacterium. If this is the case, the bacterium would not be able to survive on the LB medium without buprofezin.

**Response**: Many thanks for your comment. we agree with your opinion. In nature, *Bup_Serratia* can't survive if it only takes advantage of buprofezin, so it must obtain carbon sources from the environment. However, we demonstrate that this bacterium can utilize buprofezin to provide the sole carbon source for its growth, in turn, which can explain why the BPH infected this bacterium will increase the resistance to buprofezin. So we present the viewpoint that *Bup_Serratia* can increase host resistance to buprofezin by metabolizing buprofezin.

4) It was a great idea to examine possible involvement of Bup_Serratia in degrading buprofezin by inoculating the buprofezin-susceptible planthoppers with Bup_Serratia followed by buprofezin bioassays. However, the results would be more convincing if the authors have included an additional control of the LB medium containing a S. marcescens strain incapable of degrading buprofezin. This additional control can eliminate possible absorbance of the insecticide by the bacterial cells or the cell debris, which reduce the contact of the insect to the insecticide. This is particular helpful as the difference in survival rate between the control (the LB medium only) and the treatment is only abut 20% at 120 hours (Fig. 3D).

**Response**: Many thanks for your useful suggestions. Although we were not able to source a S. marcescens strain incapable of degrading buprofezin we have conducted an additional experiment along the same lines. Specifically, we tested the change of susceptibility to buprofezin after the NIS strain of BPH was infected by *E. coli*. The result showed that there was no difference in sensitivity of *E. coli* infected BPH and uninfected BPH to buprofezin. Please see the lines 363-365 and Fig S2.

5) Fig. 1B shows that the resistant strain became more susceptible (the survival rate decreased from about 80% to 40%) when the insect was treated with tetracycline. I was wondering if this relatively small change can completely explain over 40-fold of resistance. Are there any other resistance mechanisms involved in buprofezin resistance in this resistant strain, and/or is the growth inhibition of the bacteria after treated with the antibiotic under the experimental conditions complete. The authors might want to clarify or discuss this.

**Response**: Many thanks for your suggestion. We don’t believe that symbiotic bacteria can completely explain the resistance of the NIB strain to buprofezin. We agree with the reviewer that it would be interesting to explore other mechanisms of resistance in the NIB strain, however, this would involve RNAseq analysis and potentially functional charaterisation of candidate P450s/mutations which might take 18 months or more to conduct. Thus while interesting we feel this would be outside the scope of the current manuscript which aimes to investigate mechanisms of resistance that are not encoded by the insect host genome. However, we have added text to the discussion (lines 595-599) acknowledging that it would be interesting to look into mechanisms of resistance in the NIB strain in future (and how these inteact with symbiont mediated resistance).

Part III – Minor Issues: Editorial and Data Presentation Modifications

Reviewer #1:

1) The manuscript needs to be further polished to correct some English issues. For example, on Line 24, a symbiotic "bacteria" should be changed to a symbiotic "bacterium". On Line 27, "sensitive" should be changed to "susceptible". On Line 59, the sentence "Among the most damaging of these pests and pathogens are insects" is grammatically incorrect.

**Response**: Many thanks for your suggestion. We have corrected these errors.

2) The statement on Lines 41-43 is biased regarding different resistance mechanisms. Specifically, the alteration of the expression of detoxification enzymes is not caused by gene mutations. There are some examples that the gene mutations may result in more efficient enzymes for degrading insecticides.

**Response**: Many thanks. We think the mutation in promoter sequences that leads to the alteration of expression of detoxification enzymes also belongs to genome variation. Of course, we agree with your opinion that the mutation in the coding region of detoxification enzyme genes will result in more efficient enzymes for degrading insecticides. So we have added text to acknowledge this, please see line 43.

3) On Line 51, I suggest to change "microbe x host insect x environment interactions" into "microbe-host insect-environment interactions".

**Response**: Many thanks for your suggestion. We have made this change.

4) On line 336, what do "Shannon and Simpson indices" exactly tell us? Please explain the differences.

**Response**: Many thanks for your question. Shannon and Simpson indices comprehensively reflect the evenness (a measure of the relative abundances of species within a community) and richness of bacteria species. The larger the value of Shannon, the more evenness. The larger the value of Simpson, the higher the diversity. We have added text to this section of the manuscript to make this clear.

5) Please describe the experiments of treating the NIS strain with the LB media containing Bup_Serratia. For example, how did the authors treat the insects and what was the time interval between the treatments of Bup_Serratia and buprofezin.

**Response**: Many thanks for your question. As described in methods of “Infection of *N. lugens* with *Bup_Serratia*”. About 200 adult *N. lugens* from the NIS strain were transferred to 15-day-old rice seedlings that were planted in a 1000 mL glass breaker on a layer of absorbent cotton. After five days adult *N. lugens* will be removed from the breaker. 100 mL of *Bup_Serratia*, which was cultured in LB medium and grown to an OD_600_ of 0.8-1.0, was poured into the beaker on the first day that *N. lugens* were transferred. *Bup_Serratia* was replaced with fresh bacteria every three days, which can make the rice seedling absorb the *Bup_Serraria* and transmit it to eggs of BPH. After approximately thirteen days, the majority of the eggs had hatched into nymphs. These nymphs were then transferred to fresh rice seedlings that were free from *Bup_Serratia* for continuous rearing for five days until they reached the third instar, after which they were used for buprofezin susceptibility testing as described above. LB medium without *Bup_Serratia* was used as a control.

6) Fig3. D and E appear to be incorrectly labeled. Based on the data, E should be D while D should be E.

**Response**: Many thanks for pointing out this mistake. We have corrected it.

Reviewer #2:

1. The strains (NIS or NIB) used in each panel should be indicated in the Fig 1 for clarity.

**Response**: Many thanks for your suggestion. We have added this information.

2. I think the authors did not test the original G0 strains with antiboitics or 16S sequencing, so the buprofezin resistance may be associated with symbiont bacteria, but not "the increase of buprofezin resistance". It will be also useful if the authors can give the rationale about why they chose this Yunan strain.

**Response**: Many thanks for your suggestion. We have change the text. Regarding why we chose the Yunan strain, there is no special cause, we just selected a field strain of BPH to perform this project.

3, Fig 3D, what is SS? I prefer full name here. Besides, the figure legends for panel D and E should be exchanged.

**Response**: Many thanks for pointing out this mistake. We have corrected it.

4, From line 372 and in discussion, there is currently no biochemical or genetic confirmation regarding these candidate genes, and their amino acid sequence identity to known BfzA-C is not very high, thus the authors should exercise caution when drawing their conclusions.

**Response**: Many thanks for your suggestion. We have moderated the conclusion made in the discussion accordingly.

5, The detection of Bup_Serratia from field planthopper populations is important to show the ecological relevance of this symbiont-mediated resistance. As a result, are these strains more resistant than NIS strains against buprofezin?

**Response**: Many thanks for your question. Yes, all field planthopper populations are much more resistant than the NIS strain to buprofezin. It has been reported that the BPH from the field has developed high levels of resistance to buprofezin since 2013 in China [1]. However, the specific field strains collected were not tested against buprofezin in the current study – we hope to conduct such assays in a future study.

6, Line 81, the authors mentioned that there are other resistant mechanisms such as overexpression of P450 and target-site mutation, therefore, they should also check these genetic characteristics in both sensitive NIS strain and resistant NIB strain (because these two strains were collected from two distant locations). Such results will be helpful to show the precise contribution of symbionts to buprofezin resistance.

**Response**: Many thanks for your suggestion. We agree with the reviewer that it would be interesting to explore other mechanisms of resistance in the NIB strain, however, this would involve RNAseq analysis and potentially functional charaterisation of candidate P450s/mutations which might take 18 months or more to conduct. Thus while interesting we feel this would be outside the scope of the current manuscript which aimes to investigate mechanisms of resistance that are not encoded by the insect host genome. However, we have added text to the discussion (lines 595-599) acknowledging that it would be interesting to look into mechanisms of resistance in the NIB strain in future (and how these inteact with symbiont mediated resistance).

1.	Zeng B, Chen F-R, Liu Y-T, Di G, Zhang Y-J, Feng Z-R, et al. A chitin synthase mutation confers widespread resistance to buprofezin, a chitin synthesis inhibitor, in the brown planthopper, *Nilaparvata lugens*. J Pest Sci. 2023;96:819-32. doi: 10.1007/s10340-022-01538-9.
